# Giant cell tumor of distal ulna treated using en-bloc resection combined with extensor carpi ulnaris and flexor carpi ulnaris tendon stabilization: A case report

**DOI:** 10.1016/j.ijscr.2021.01.065

**Published:** 2021-01-20

**Authors:** Iman Solichin, Windi Martika, Rio Wikanjaya

**Affiliations:** a Department of Orthopaedics and Traumatology, Purwokerto Orthopaedics Hospital, Purwokerto, Central Java, Indonesia; b Department of Orthopaedic and Traumatology, Dr. Cipto Mangunkusumo National Central General Hospital – Faculty of Medicine, Universitas Indonesia, Jakarta, Indonesia

**Keywords:** Giant cell tumor of bone, GCT, Distal ulna, En-bloc resection

## Abstract

**Introduction:**

Giant Cell Tumor (GCT) is a form of bone tumor which is rare, benign, and locally invasive. To date, there have not been many case reports regarding cases of GCTs on the distal ulna which made the optimum strategy in management remain controversial. In some reported cases, the patient was treated with wide excision followed by reconstructive procedure resulting in ulnar translation of the carpal bones and dynamic convergence of the ulna towards the radius.

**Presentation of case:**

We documented a case of 29-year-old male with distal ulna GCT, treated with en-bloc resection combined with extensor carpi ulnaris and flexor carpi ulnaris tendon stabilization. The key objectives of GCT treatments are to avoid local recurrence with sufficient resection and to maintain the function of the limbs. Specific treatment options have been suggested for en-bloc resection with or without the need for ulnar reconstruction or stabilization, even prothesis. In this case, we excised the distal portion of the ulna with some soft tissue procedure for added stability.

**Clinical discussion:**

Three weeks after the surgery, the patient was able to perform wrist flexion and extension, fingers abduction, adduction, and opposition with slight limitation. The DASH and PRWE score had improved gradually within 3 weeks and 6 months after the surgery.

**Conclusion:**

In the subsequent evaluation after six months of the surgery, the patient achieved full restoration of forearm function without any limitation.

## Highlights

• Giant cell tumor (GCT) especially in distal ulna is a rare and benign neoplasm, but locally invasive tumor.
• En-bloc resection combined with extensor carpi ulnaris and flexor carpi ulnaris stabilization was performed to excise GCT of distal ulna.
• This procedure gives full restoration of forearm function without any limitation and produces excellent DASH score.

## 1 Introduction

Giant cell tumor (GCT) is a rare, benign, but locally invasive tumor. The tumor develops between the ages of 20 and 40 years old, with a higher occurrence in women. GCT of bone typically occurs at long bone epiphysis, such as femur, tibia, humerus and radius [1]. The pathology is typically portrayed by mononuclear histiocytic cells with multinucleated giant cells resembling osteoclasts and neoplastic stromal cells that are the predominantly proliferating cell population [2]. The most common sites for GCT are at the end of metaphyseal region of long bone such as distal radius and femur, proximal humerus and ulna. Rarely, GCT forms at the distal end of the ulna which accounts for 0,45–3,2% of primary bone GCT [2,3].

The prevailing issue in GCT management is local recurrence following surgical treatment: 27 65% after isolated curettage; 12 27% after curettage with adjuvants such as high-speed burr, phenol, liquid nitrogen or polymethylmethacrylate; and 0 12% after en-bloc resection [4]. To this date, the optimal treatment strategies for distal ulna and radius GCTs remain controversial. Curettage (intralesional excision) protects the joint, but it has a relatively high probability of local recurrence when it is conducted concurrently with surgical adjuvants, including liquid nitrogen, phenol or cement. On the other hand, en-bloc resection of GCT provides lower rates of recurrences. However, it potentially sacrifices the joint which later requires a major reconstruction. Therefore, the effect on tumor recurrence and postoperative wrist function of various surgical modalities for GCT of distal ulna remains unclear [1,2,5].

We recorded a case of 29-year-old male with distal ulna GCT treated by en-bloc resection combined with extensor carpi ulnaris (ECU) and flexor carpi ulnaris (FCU) stabilization. The patient achieved painless wrist joint movement within 3 weeks and full functional restoration of forearm within 6 months after the surgery. This report has been reported in line with the SCARE criteria [6].

## 2 Case presentation

**Fig. 1:**
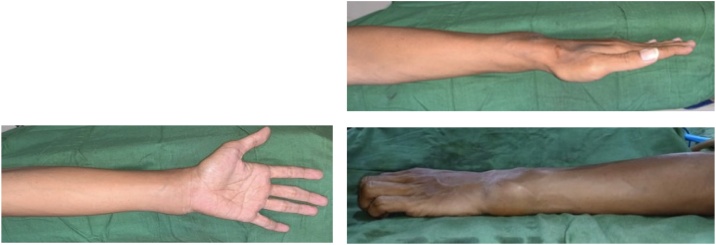
The clinical appearance of the mass on the left wrist.

A 29-year-old male with a left wrist mass which he felt for the last 6 months before admission. The swelling began to appear noticeably when the patient fell to his left side while playing soccer. After the fall, the patient complained of pain in the left wrist and was brought to a traditional bone setter where he received several massage therapies. The mass was said to had been as big as marble at first. However, the patient did not seek for any medication until several months prior to admission. At that moment, the patient complained that the mass grew as big as baseball, and he started to feel pain. The patient was right-handed (Fig. 1).

The general state was within normal limit. During the local examination of the left wrist, there was a visible mass in the distal ulna sized 9 × 7 × 3 cm. The mass was hard, immobile, and painful upon palpation (VAS 1–2). The patient was still able to move his left wrist, but the range of movement was limited due to the pain. The DASH score and PRWE score were 46.67 and 45.5 respectively, which indicated severe disability.

**Fig. 2:**
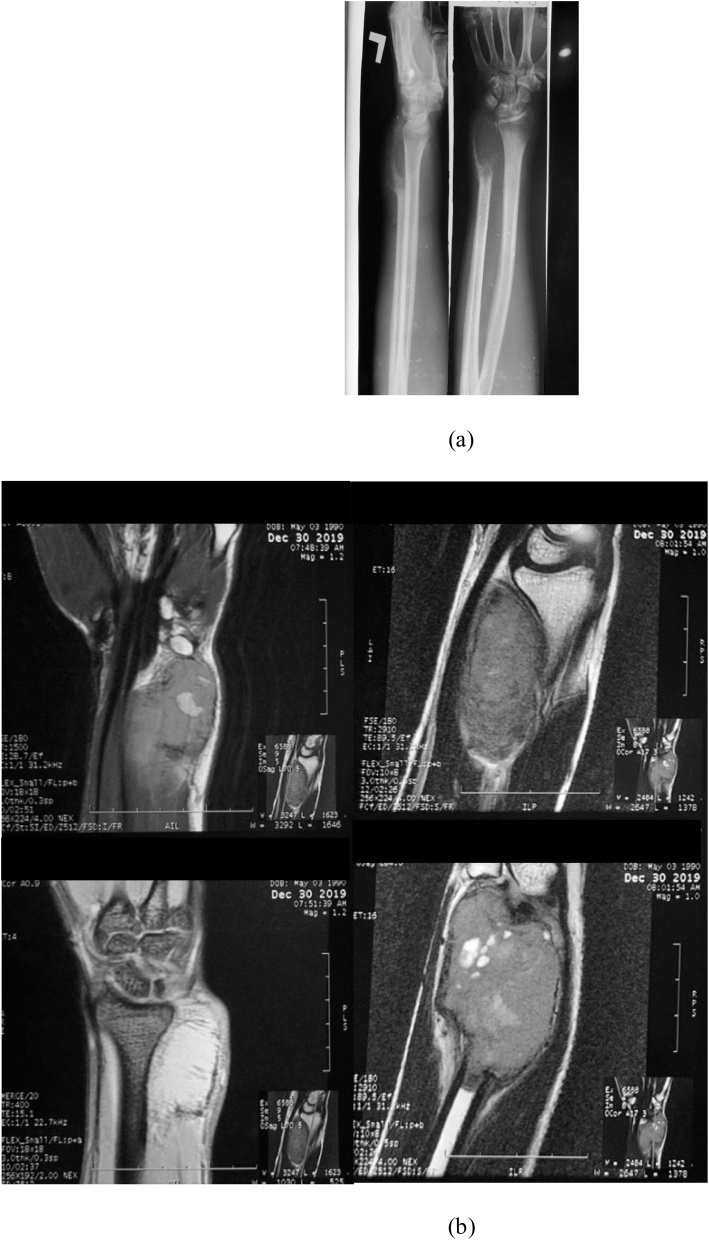
(a) Preoperative plain X-ray showed an expansile, multi-lobular, and radiolucent lesion with a clear margin in the distal end of the left ulna (b) MRI of the left wrist.

**Fig. 3:**
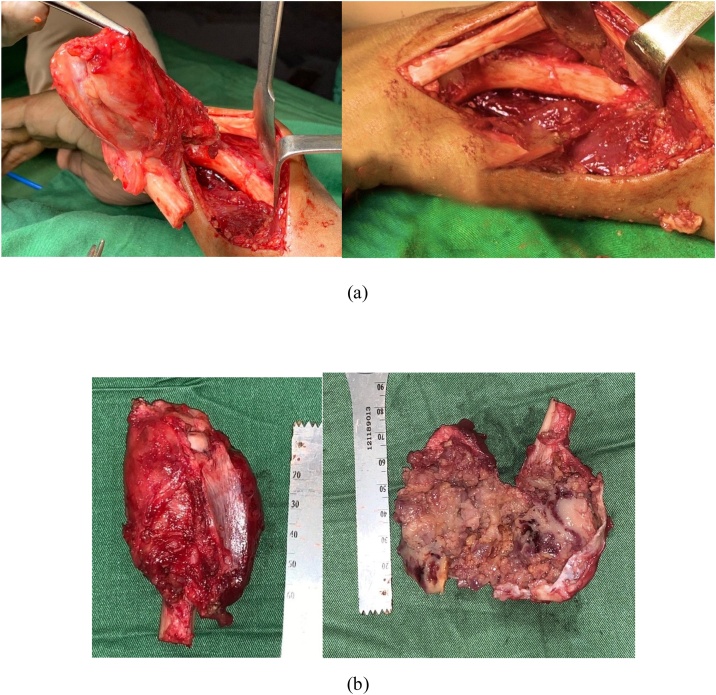
(a) Intraoperative clinical photograph showing GCT of distal ulna: resected specimen showing complete excision of distal ulna. (b) Tumor of the left wrist after en-bloc resection: cut section of the specimen showing expansile lobulated mass.

**Fig. 4:**
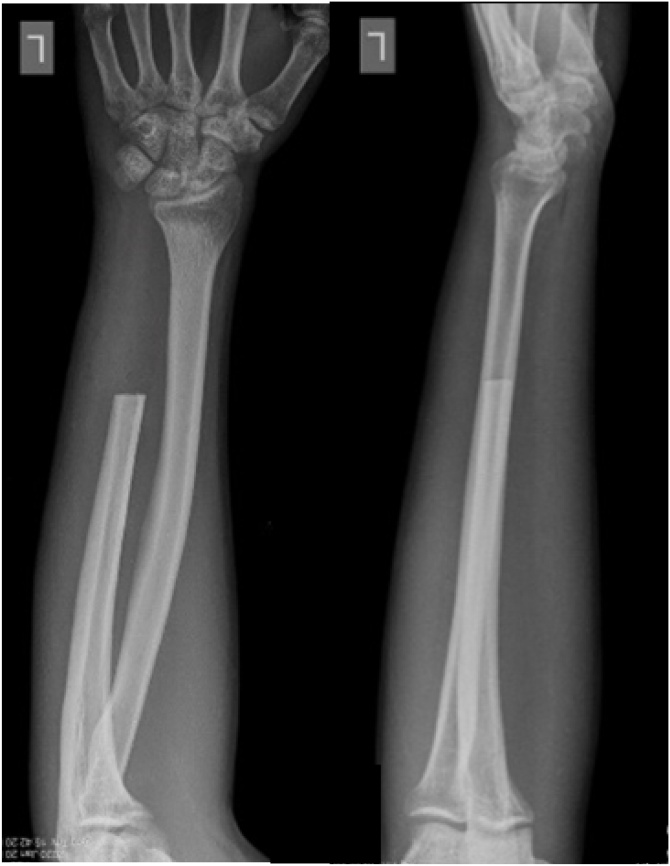
Postoperative radiograph after 3 weeks.

Conventional radiography revealed a multi-lobular and radio-lucent area with a clear margin in the distal ulna (Fig. 2). There was no sign of fracture, but an osteolytic area in the ulnar metadiaphysis was found. Chest X-ray showed no sign of pulmonary metastases. Wrist MRI detected a lesion measured 8 cm in length and 4 cm in width, expanding and partially destroying the thin cortex. We sent the core biopsy speciment to histopathologic department and showed multinucleated giant cell with more than 20 nucleus. The patient was treated with en-bloc resection surgery by fully experienced orthopaedic surgeon who has more than 35 years in managing this special case (Fig. 3, Fig. 4).

**Fig. 5:**
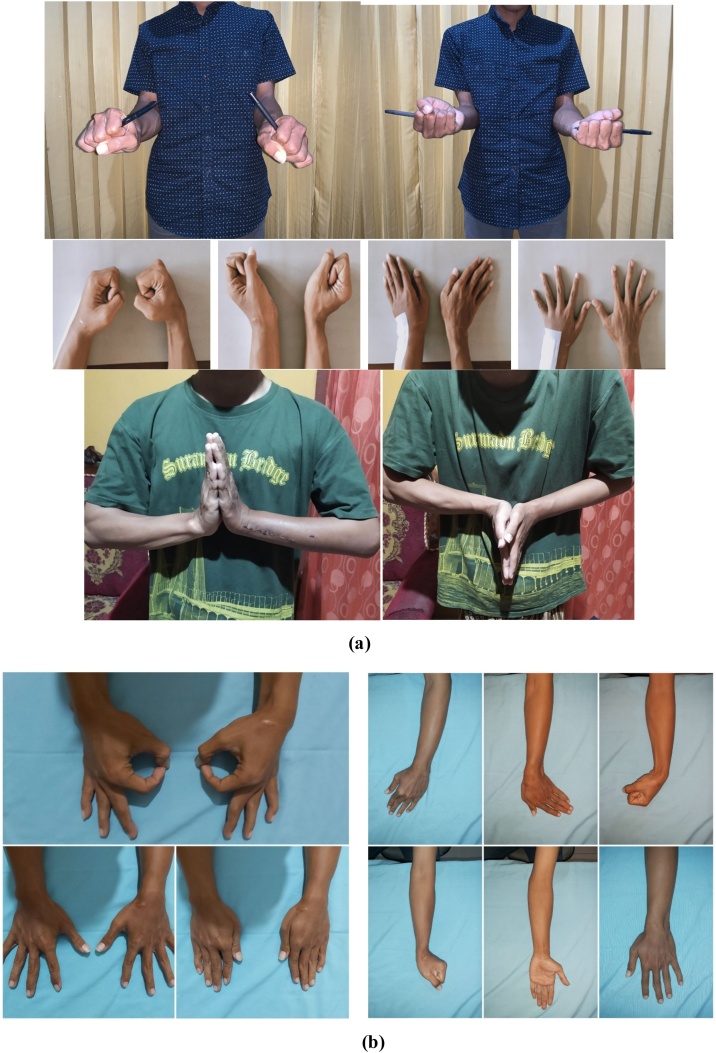
(a) Postoperative evaluation after 3 weeks post wide resection surgery (b) Postoperative evaluation after 6 months of surgery.

Three weeks after the surgery, the pain on the wrist has reduced significantly and the patient was able to do abduction, adduction, flexion, extension, opposition as well as the normal side. The DASH score and PRWE score were 13,33 and 8 respectively which indicated mild disability (Fig. 5).

The DASH score was 4.17 after six months of surgery. The PRWE was excellent with score of 2. The patient might return to his normal daily activity without any disturbances. We encouraged the patient to take routine examination every six months for monitoring signs of recurrency as GCT has a high recurrency rate.

## 3 Discussion

Giant cell tumor of bone (GCT) is a rare benign neoplasm with locally aggressive behavior, accounting for approximately 5% of all primary bone tumors. The reported annual incidence ranges from 0.65 to 1 cases per million population. Such condition affects people between the age of 20 and 40 years and was found more common in women than in men [7,8]. Distal femur, proximal tibia, distal radius, and proximal humerus are the most common sites for bone GCT in decreasing order of frequency. Bone GCT in the distal ulna is very rare, with a reported incidence of merely 0.45–6 per cent [9].

Various treatment options have been proposed for GCT, including intralesional curretage, curretage and filling with bone graft, cavity cryotherapy after curretage, phenol after curretage, radiation, methylmetacrylate cement insertion in the cavity after curretage, resection followed by allograft, en-bloc resection with or without ulna reconstruction or stabilization [9].

The main objectives of GCT treatment are to prevent local recurrence with adequate resection and to maintain limb function. Intralesional procedure is the treatment of choice for this benign aggressive lesion when bone and joint structure is well preserved. However, many studies have shown high rates of recurrency [10]. There is no consensus on the best treatment for bone GCT in the distal end of the ulna due to its rareness. Distal ulna aids the rotation of the forearm. It also facilitates grip strength and maintains the link between the carpus and the distal part of the radius.

Two treatment options are available to treat benign aggressive distal ulna tumors, including GCT. Curettage may be the first treatment option to be used when GCT is limited to bone cortex. Distal ulna resection is another option and it is performed when the tumor is not confined to the cortex, or in the case of a soft tissue component, a pathological fracture, or an unsuccessful previous surgery [11]. Darrach et al. [12] reported that the distal ulna can be excised sacrificing function and indicated its excision for degenerative conditions.

In our case, we removed the distal part of the ulna without any biological reconstruction, such as bone grafting. Another report suggested that there was risk of having a decrease function of the forearm after the excision, which was not evident in our case. Our patient was able to achieve painless wrist joint movement within three weeks after the surgery, including flexion, extension, radial deviation, ulnar deviation, pronation, and supination. The DASH score was 13.33 (mild disability) three weeks after the surgery. This result was consistent with a study conducted by Cooney et al. which stated a 75% excellent result after distal ulnar GCT excision. Six months after the surgery, the patient came to our outpatient clinic with satisfactory result without any sign of ulnar nerve palsy and any limitation of forearm function. The final DASH score (six months after the surgery) was 4.7 and it had reduced significantly from the initial DASH score that was 46.67 (severe disability). The PRWE was excellent in our patient at six months follow-up. This indicates that there was no significant wrist disturbance in daily activity. Both pain and function evaluation were great.

On the other hand, numerous authors have documented that the en bloc resection of the distal ulna could be predicted to fail due to the dorsal translation of the ulna at the resection site during pronation. Excessive resection of the distal ulna usually results in painful stump or clicks instability. In our case, it is important to measure and correctly identify the margin of the tumor and the area of excision. With better precision, the forearm functionality can be achieved [11].

## 4 Conclusion

En-bloc resection alone may become the treatment of choice for GCT of the distal ulna as the improvement of DASH score occurred within three weeks and six weeks after the surgery in our case. Further studies are required to examine the effectiveness of wide excision only of the distal ulna as the treatment for not only GCT, but also other primary bone tumors involving the distal ulna.

## Declaration of Competing Interest

The authors report no declarations of interest.

## Funding

The authors report no external source of funding during the writing of this article.

## Ethical approval

Ethical approval was not required in the treatment of the patient in this report.

## Consent

Written informed consent was obtained from the patient for publication of this case report and accompanying images. A copy of the written consent is available for review by the Editor-in-Chief of this journal on request.

## Author contribution

Iman Solichin contributes in the study concept or design, data collection, analysis and interpretation, oversight and leadership responsibility for the research activity planning and execution, including mentorship external to the core team.

Windi Martika contributes to the study concept or design, data collection and writing the paper.

Rio Wikanjaya contributes to the data collection and writing the paper.

## Registration of research studies

Not applicable.

## Guarantor

Iman Solichin.

## Provenance and peer review

Not commissioned, externally peer-reviewed.

## Disclaimer

No patient or author details are included in the figures.
